# Epigenetic Clocks and Their Prospective Application in the Complex Landscape of Aging and Alzheimer’s Disease

**DOI:** 10.3390/genes16060679

**Published:** 2025-05-30

**Authors:** Annamaria Cerantonio, Beatrice Maria Greco, Luigi Citrigno, Selene De Benedittis, Antonio Qualtieri, Raffaele Maletta, Alberto Montesanto, Giuseppe Passarino, Patrizia Spadafora, Francesca Cavalcanti

**Affiliations:** 1 Institute for Biomedical Research and Innovation, National Research Council (IRIB-CNR), 87050 Mangone, CS, Italy; beatricemaria.greco@irib.cnr.it (B.M.G.); luigi.citrigno@cnr.it (L.C.); selenedebenedittis@cnr.it (S.D.B.); antonio.qualtieri@cnr.it (A.Q.); patrizia.spadafora@cnr.it (P.S.); francesca.cavalcanti@cnr.it (F.C.); 2 Department of Biology, Ecology and Earth Sciences, University of Calabria, 87036 Rende, CS, Italy; alberto.montesanto@unical.it (A.M.); giuseppe.passarino@unical.it (G.P.); 3 Regional Neurogenetic Centre (CRN), Department of Primary Care, ASP Catanzaro, 88046 Lamezia Terme, CZ, Italy; raffaelegiovanni.maletta@asp.cz.it; 4 Association for Neurogenetic Research (ARN), 88046 Lamezia Terme, CZ, Italy

**Keywords:** epigenetics, DNA methylation, epigenetic clocks, Alzheimer’s disease, aging, cognitive decline

## Abstract

Nowadays, scientists are making efforts to elucidate the mechanisms involved in the phenotypic changes underlying the aging process in order to develop favorable therapeutical interventions. Epigenetic modifications, in particular DNA methylation, play a crucial role in the aging process, and this parameter has been used to set epigenetic clocks, algorithms that predict an individual’s biological age based on a defined set of CpGs. In this review, we focus on the most recent literature to discuss the use of epigenetic clocks in the context of cognitive decline and dysregulation of Alzheimer’s disease (AD)-related gene expression. We have summarized all published scientific papers in which epigenetic clocks have been applied to measure age acceleration in blood and brain specimens from patients affected with AD. Progressive age acceleration, consistent with a specific DNA methylation signature, was observed in patients affected by AD, and it was correlated with the onset of complex diseases, mitochondrial alterations, dementia and cognitive decline, even in the early stages of these conditions. The use of epigenetic clocks might be a valuable biomarker to enable an earlier identification of ideal measures to reverse modifications caused by aging and to mitigate multiple aspects of disease/aging mechanisms.

## 1. Introduction

### 1.1. Aging and Underlying Mechanisms of Age-Related Declines

Aging is a complex biological process related to both the lifespan and healthspan of subjects. This event has a high inter-individual variability and it is almost always influenced by genetic, environmental, and lifestyle factors. Individuals of the same chronological age, in fact, may show differences linked to the effect of diseases, morbidity, mortality, and physical or cognitive decline representative of biological age [1].

Since birth, we gradually begin to age and therefore cells, tissues and organs are continuously in a state of irreversible pathophysiological decay [2].

One of the initiating events responsible for the aging process is the accumulation of DNA mutations [3]. DNA alterations are caused by physical, chemical, and biological agents, but the accumulation of DNA replication errors and damages caused by Reactive Oxygen Species (ROS) are important key factors. DNA alterations include mutations, translocations, telomere shortening and gene disruption due to the integration of viruses or transposons [4].

However, cells have different DNA repair mechanisms able to repair damage, such as Mismatch Repair (MMR) and Base Excision Repair (BER), which are responsible for removing mismatched and damaged bases, respectively [5].

During aging, these protective mechanisms do not work as well as at the beginning of one’s life [4]. DNA alterations must be repaired to guarantee a normal homeostasis; otherwise, genetic damage will be a dangerous consequence of genome instability and it will result in premature aging. Aging is also characterized by epigenetic alterations such as changes in DNA methylation patterns, histone modifications, and chromatin remodeling [4]. It is easy to understand that many different processes are involved in aging, like loss of proteostasis, mitochondrial dysfunction, immunosenescence and stem cell exhaustion [6].

Proteostasis is the way through which homeostasis is preserved owing to the activity of chaperones and proteolytic systems, consisting of two main components: lysosome autophagy and the ubiquitin–proteasome system. Organisms are always susceptible to progressive alterations in protein homeostasis but during aging this apparatus fails to execute its ordinary tasks; so, an accumulation of unfolded, misfolded or aggregated proteins occurs and these events lay the foundation for the onset of many diseases [7].

Several studies demonstrated that the loss of autophagic activity could be ascribed to DNA methylation by the direct modification and silencing of autophagy-related genes [8].

The use of DNA methylation inhibitors could positively restore phenotypic changes associated with aging by reactivating autophagy-related signaling molecules [9]. Concurrently, studies on small molecules able to activate methylated genes have become relevant. Some of these molecules exhibited a direct role in targeting the p38 Mitogen-Activated Protein Kinase (MAPK) pathway, thus activating genes previously silenced by the action of DNA methylation [10].

Other pro-methylating molecules displayed the capability to bind the Methyl Transferase-like 3 (METTL3)/METTL14/Wilm’s Tumor-1-Associated Protein (WTAP) complexes, thus increasing the cellular levels of N6-methyladenosine (m6A), whose deficiency has been involved in several diseases such as cancer, neurodegeneration and aging [11].

Mitochondria are membrane-bound cell organelles that contain their own DNA, which encodes for tRNAs, rRNAs, and some mitochondrial proteins. Mitochondria are considered the energetic resource of mammals because they produce most of the chemical components required to perform all the biochemical reactions within cells. Among the functions carried out by mitochondria, the most significant is the handling of macromolecules, such as carbohydrates and fatty acids, with the purpose to synthesize ATP during Oxidative Phosphorylation (OXPHOS) [12].

The accumulation of damaged mitochondria during the natural aging process causes an increasing production of ROS and a general dysfunction of these organelles, which are lacking in the DNA repair mechanisms possessed by nuclear DNA. All these factors are therefore capable of triggering a stress response in the endoplasmic reticulum, compromising the electron transport chain processes and, subsequently energy production [13].

It has been demonstrated that several mitochondrial methyl transferases modify their expression during aging, highlighting the key role of hypermethylated regions in the landscape of degenerative diseases. All these findings suggest that mitochondria could be considered a potential target for extending healthspan [14].

Stem cells are important for the correct homeostasis of tissues and, at the same time, for regular cell turnover in humans and animals. The depletion of stem cells leads to a progressive qualitative and quantitative loss of the regenerative capacity of progenitor cells and a consequent decrease in performance in tissue maintenance and repair. Stem cell deficiency is characteristic of aging and this event might be caused by the exhaustion of the self-renewal activity of cells or by the high rate of apoptosis/senescence induced by continuous exposure to cellular stress [6,15].

Stem-cell niches undergo a quiescent state during their life. For this reason, stem cells in aged tissues are subjected to long-term exposure to endogenous and exogenous genotoxic sources, resulting in the accumulation of alterations and a reduction in the rate of repair in response to DNA damage [16].

Immunosenescence, typical for elderly populations, is defined as an age-related immune dysfunction characterized by changes in the production of several immune components with a high prevalence of inflammatory factors [6]. Several studies have focused on the detection of molecular changes typical for immunosenescence and the hallmarks of this condition include: a reduced ability to respond to new antigens; accumulation of memory T cells; a persistent and chronic low-grade inflammation termed “inflamm-aging” [17].

Immunosenescence is characterized by thymic involution, altered T and B cell responses, altered naïve/memory ratio, increased serum IgG and IgA levels and a poor response to new microbial antigens, including vaccines [18].

### 1.2. Epigenetics and Its Role in the Landscape of Aging

The term epigenetics was defined in the 1940s by Conrad Waddington as “the branch of biology which studies the causal interactions between genes and their products that bring the phenotype into being”. In detail, epigenetics is focused on the interaction between environment and genetic expression and this interconnection is widely investigated in the study of aging. Gene expression is influenced by molecular mechanisms such as DNA methylation and histone modifications [19]. Even if these two mechanisms are carried out by different chemical reactions and require the action of different enzymes, the biological purpose of these systems is the modulation of gene expression [20].

DNA methylation consists of the addition of a methyl group to carbon 5 of a cytosine occurring at CpG dinucleotides. This reaction is catalyzed by a group of enzymes called DNA methyltransferases (DNMTs) that specifically transfer a methyl group from S-adenosyl-methionine (SAM) to position 5 of cytosine. The most relevant DNMTs are DNMT1, DNMT3A, and DNMT3B.

DNMT1 is the most abundant enzyme and the key methyltransferase for the maintenance of DNA methylation during DNA replication, guaranteeing the correct transmission of epigenetic methylation traits to progeny cells [21].

DNMT3A and DNMT3B are mainly involved in de novo DNA methylation events during embryo development and germ cell differentiation, but they also play a crucial role in the occurrence of DNA methylation at sites other than CpG sequences, such as methylation at cytosines followed by adenine (CpA), thymine (CpT) or another cytosine (CpC) [22].

This phenomenon, called non-CpG methylation, was detected in pluripotent stem cells and oocytes, but also in neurons [23].

It has been reported that non-CpG methylation could regulate mRNA expression at gene-specific levels, and differential CpG and non-CpG methylation events have been detected in both mitochondrial and nuclear DNA of human brain tissues of patients affected by neurodegenerative disorders [24,25].

In general, aging causes both genome-wide demethylation and hypermethylation events at specific sites; thus, studying these mechanisms might be useful to determine biological age and to develop reliable epigenetic clocks. Moreover, DNA methylation is involved in a large number of key processes like genomic imprinting, X-chromosome inactivation and transcriptional repression, especially for the inability of transcription factors to bind promoter regions.

Histone modifications include lysine methylation, arginine methylation, lysine acetylation and serine phosphorylation. These types of modifications not only cause an alteration in the interaction between DNA and histones but also reduce the activation of several canonical genes [6].

The existence of a complex relationship between DNA methylation patterns and the aging process has been hypothesized and significant changes in methylation signature have been reported. These changes are caused by hypomethylation, observed in repetitive DNA sequences and leading to genomic instability and hypermethylation at specific CpG islands in gene promoters, resulting in gene silencing in adult tissues [26].

Moreover, regarding complex diseases, there are many environmental factors whose exposure can promote epigenetic changes, such as smoking, infectious pathogens, outdoor pollutants, indoor allergens and heavy metals [27].

### 1.3. Epigenetic Clocks and Their Advancements in Measuring Aging and Age-Related Damages

Epigenetic clocks are age prediction models that rely on the methylation levels of specific genetic loci to determine biological age. There is a significant difference between biological and chronological age, which is marked by age acceleration [28]. Chronological age can be defined as the numbers of years after birth, while biological age is defined as physiological, organismal or phenotypic age. However, the latter definition is not entirely comprehensive, especially since many genetic or environmental factors can interfere with it. In any case, biological age (also called epigenetic age) seems to be strongly related to the biological state of the individual; so, it might differ in several ways even among people with the same chronological age [29].

Epigenetic clocks are categorized into four generations. The first-generation epigenetic clocks are represented by the clocks developed by Horvath and Hannum.

Horvath was the first author to develop a multi-tissue age predictor by analyzing 353 CpG sites in 51 different tissues and to demonstrate that tissues may have an accelerated age due to diseases such as cancer [30].

Hannum developed an epigenetic clock using 71 epigenetic biomarkers in 656 blood samples. This model can be useful for determining age with an error of less than 3–9 years, and interestingly, when tested on different types of tissues, it showed that the aging rate differs between normal and cancerous tissues, respectively. These clocks are very accurate; so, if they register a discrepancy with chronological age, there is a high probability of death [31]. However, data used to set up these epigenetic clocks often lack accuracy, especially when applied to older age groups or age-related diseases [32].

The second-generation epigenetic clocks are PhenoAge and GrimAge. Their goal is to predict all-cause mortality and morbidity related to epigenetic modifications. Furthermore, their applications seem to be feasible in several tissues or cells.

PhenoAge was developed using blood samples, 513 CpG sites and 10 clinical biomarkers such as albumin, creatinine, glucose, C-Reactive Protein (CRP), lymphocyte percent, mean cell volume, red cell distribution width, alkaline phosphatase, white blood cell count and age [19].

GrimAge was designed to combine DNAm-based surrogate biomarkers for health-related plasma proteins and smoking-pack-years, as well as sex and chronological age. This epigenetic clock shows a high association with key “hallmarks of aging,” such as cellular senescence [33].

Third-generation epigenetic clocks include Dunedin Pace of Aging Methylation (DunedinPoAm38) and later, the DunedinPACE, used to evaluate how epigenetic age changes across the landscape of complex diseases and frailty [34].

This measure is based on fluctuations in phenotypic measures, including 19 biomarkers of “health status”. These are: body mass index, waist–hip ratio, glycated hemoglobin, leptin, blood pressure, cardiorespiratory capability, forced vital capacity ratio, forced expiratory volume, total cholesterol, triglycerides, high-density lipoprotein, lipoprotein(a), apolipoprotein B100/A1 ratio, estimated glomerular filtration rate, blood urea nitrogen, high-sensitivity CRP, white blood cell count, mean periodontal attachment loss, and number of dental caries affected tooth surfaces. An algorithm that combines all these parameters is able to discriminate among subjects with different rates of aging, where people with “accelerated aging” show a higher risk of developing complex diseases or of experiencing premature death [35].

Fourth-generation epigenetic clocks (causal clocks), derived by Mendelian Randomization studies, focus on the analysis of CpG sites causally linked to the aging process and to detrimental and adaptive methylation changes. The source from which these sites were obtained considers healthspan, father’s and mother’s lifespan, exceptional longevity, frailty index and self-rated health. Subsequently, data obtained by these informations have been used to classify CpG sites as upregulated in healthy agers, downregulated in less healthy agers (both linked to an adaptation to aging) or downregulated in healthy agers and upregulated in less healthy agers (markers of aging-related damage). These results have been converted into three mathematical algorithms: CausalAge (age-related causal sites), AdaptAge (sites representative of adaptation to aging) and DamAge (sites associated with age-related damage) [36]. Epigenetic clocks, as biomarkers of aging, could be useful for predicting age-related disorders such as dementia [37].

### 1.4. Alzheimer’s Disease as a Consequence of Aging Process

Alzheimer’s disease (AD) is a progressive neurodegenerative disorder and it represents the world’s leading form of dementia. It is estimated that 131.5 million people will be affected by AD by 2050 if no new pharmacological therapies become available [38].

At the neurological level, the main features of AD are the impairment of both neuronal functions and structures, the loss of synaptic connections, and neuroinflammation [39].

AD is a multifactorial disease and two forms of this disorder exist: Sporadic (SAD) and Familial (FAD). SAD represents the most common form, but the exact cause remains unclear. It is characterized by late onset (onset > 65 years) and for this reason, it is also called late-onset Alzheimer’s disease (LOAD). FAD, instead, shows an early onset (<65 years) and it is called early-onset Alzheimer’s disease (EOAD). EOAD generally follows a Mendelian inheritance due to mutations identified in the *APP*, *PSEN1* and *PSEN2* genes encoding the Amyloid Precursor Protein (APP) and the two subunits of γ-secretase (PSEN1, PSEN2), respectively. In addition, several risk factors such as age, gender, level of education, cardiovascular diseases, diabetes, infections and environmental factors seem to contribute to AD onset [40].

Brain tissue from deceased AD patients is characterized by the presence of β-amyloid plaques and tau protein hyperphosphorylation and aggregation into neurofibrillary tangles (NFTs). Aβ peptides originate from APP, a ubiquitous transmembrane protein mainly expressed in the central nervous system as the product of two proteolytic enzymes: β-secretase and γ-secretase (amyloidogenic pathway). In physiological conditions, the proteolytic reaction is catalyzed by α-secretase (instead of β-secretase) and then by γ-secretase (non-amyloidogenic pathway) [38].

The protein tau is encoded by the Microtubule-Associated Protein Tau gene (*MAPT*), which is expressed in developing and older axons. *MAPT* isoforms can contain three (3R) or four (4R) repeat domains, which are balanced under normal conditions. When this equilibrium is altered, pathological manifestation occurs. Tau protein plays a crucial role in promoting microtubule assembly and stability. When hyperphosphorylation occurs, tau proteins are no longer bound to microtubules, leading to the deposition of NFTs and the loss of cytoskeleton connections [41].

In pathologically vulnerable brain regions of AD patients, the deposition of highly insoluble and abnormal aggregates is followed by the release of pro-inflammatory cytokines and ROS, causing neuronal death [42].

Although several risk factors are involved in the onset of this disorder, age is the main one. As we live in an aging society where life expectancy has increased, the incidence of neurodegenerative disorders has increased dramatically, representing a real problem for public healthcare. In addition, it is very challenging to make an early diagnosis to treat and manage this disease.

Several theories have been proposed to explain the pathogenesis of AD. Although the amyloid cascade hypothesis is the best described and studied, there are also the cholinergic, inflammatory, microbic and infective hypotheses.

The amyloid cascade hypothesis considers the accumulation of Aβ peptides as a crucial trigger for a series of molecular events that promote the production of hyperphosphorylated tau aggregates, the activation of neuronal death mechanisms and neurodegeneration [43].

According to the cholinergic hypothesis, AD is caused by a deficit in Acetylcholine (ACh) metabolism, which plays an important role in attention, learning and memory functions. Dysfunction of ACh-containing neurons in the brain contributes to the progressive cognitive decline observed in old age and in AD [44].

The inflammatory hypothesis assumes that AD is caused by a prolonged inflammatory state as a consequence of the accumulation of amyloid peptides and tau proteins.

Aβ species could bind Toll-like receptors and this interaction stimulates the assembly of the NOD-like Receptor Protein 3 (NLRP3) inflammasome by Nuclear Factor Kappa B (NF-kB). NLRP3, through caspase-1, converts the precursors of the inflammatory cytokines pro-IL-1β and pro-IL-18 into their active forms. IL-1β and IL-18, in turn, activate neutrophils, macrophages and other microglial cells, amplifying the inflammatory response [45].

The microbic and infective hypothesis claims that AD could be caused by a prolonged exposure to microbes or infections that results in a sustained antimicrobial response that disrupts copper-based systems that regulate neurotransmission, iron homeostasis, and respiration [46].

Several studies emphasized the role of epigenetic changes in the pathogenesis of AD. This phenomenon widely affects critical processes such as synaptic plasticity, neuroinflammation and aberrant protein aggregation. Indeed, hypermethylation of promoters and enhancers in specific genomic regions stimulates transcriptional silencing and reduced expression of genes associated with neuronal activity and memory tasks. Equally, hypomethylation at certain loci can trigger the expression of transposable elements, leading to a dysregulation of gene networks [47].

Because the interplay between DNA methylation and biological aging represents a critical risk factor for the onset of AD, the use of epigenetic clocks capable of identifying mechanisms underlying aging could therefore represent a reliable biomarker to measure the impact of DNA methylation age in AD.

## 2. Materials and Methods

This review was written based on studies published in the period from 2015 to 2024, using keywords such as AD, Alzheimer disease, cognitive decline, epigenetics, epigenetic clocks, aging biomarkers, DNA methylation clocks, and accelerated aging in the PubMed database, which described the application of epigenetic clocks in AD.

The literature of interest also had to report the clinical utility of epigenetic clocks to improve current AD diagnosis, prognosis and therapeutic interventions. Articles that did not include original research (e.g., review, opinion article or conference abstract) and in which age estimation was performed using an approach other than epigenetic clocks were excluded from this analysis.

We selected all articles that met these requirements and the most relevant data are reported in this review’s results.

## 3. Results

We summarize in Table 1 the most significant data reported in the published studies.

Levine et al. [48] carried out a study involving dorsolateral prefrontal cortex (DLPFCTX) tissues of 700 white subjects (300 of whom were diagnosed with AD) with ages ranging from 75 to 80 years old. The purpose of the authors was to examine the association between epigenetic age, AD and neuropathological markers. Epigenetic age was evaluated using the Illumina Infinium HumanMethylation450 (450 K) BeadChip. Moreover, every year participants performed cognitive tests such as Episodic Memory (EM), Working Memory (WM), Semantic Memory (SM), Perceptual Orientation (PO) and Perceptual Speed (PS) to measure Global Cognitive Functioning (GCF). A correlation between epigenetic age and chronological age at time of death was found in AD patients. Furthermore, a biweight midcorrelation showed that epigenetic age acceleration was linked to neuritic plaque deposition rate, β-amyloid load and neurofibrillary status. Examining the correlation between epigenetic age and GCF, EM, WM, SM, PO and PS, an association between epigenetic age with GCF and EM was observed. These results indicated that a one-unit drop in GCF was linked to a one-third year rise in epigenetic age and the same event was noticed for EM as well. This study indicates that EM is inversely correlated with epigenetic age, amyloid load and diffuse plaques. Furthermore, the heritability of epigenetic age acceleration, assessed by the Genome-Wide Complex Trait Analyses (GCTA) software (v1.94.1), showed reliable outputs (Table 1) [48].

Lu et al. [49] examined the rate of aging in the human brain and detected genetic loci associated with accelerated aging in different brain regions. The authors studied 1793 postmortem brain tissues from 1163 European individuals (grouped into AD and healthy controls). The samples consisted of the prefrontal cortex region (PFCTX), including the dorsolateral prefrontal cortex (DLPFCTX), cerebellum (CRBLM), frontal cortex (FCTX), pons (PONS) and temporal cortex (TCTX). The Genome-Wide Association Study (GWAS) was applied to evaluate the genetic determinants of two distinct biomarkers of brain aging: DNAm age, based on 353 CpGs from the epigenetic clock method and the proportion of neurons estimated using the Cell Epigenotype Specific (CETS) algorithm. A meta-analysis approach was used to combine the GWAS results, employing a gene set enrichment analysis to determine the molecular mechanism of brain aging. The results showed that DNAm age was highly correlated with chronological age (associated with locus 1p36.12), demonstrating the high accuracy of this epigenetic clock. Moreover, the proportion of neurons, as estimated from DNA methylation data using the CETS algorithm, exhibited a positive correlation with chronological age in the CRBLM, FCTX and PFCTX. In particular, there was a significant negative correlation between epigenetic age acceleration and the proportion of neurons in the PFCTX. After adjusting for the proportion of neurons, the AD condition was found to be significantly associated with epigenetic age acceleration in the PFCTX. Gene sets identified by GWAS of epigenetic aging in the PFCTX included genes associated with cognitive decline, dementia, and AD, demonstrating a genetic overlap between neurodegeneration and epigenetic age acceleration (Table 1) [49].

McCartney et al. [50] investigated the association between different AD risk factors and age accelerators (smoking, body mass index, high blood pressure, cholesterol) in a cohort of 5100 Scottish individuals recruited for a study called Generation Scotland: the Scottish Family Health Study [50,51]. This cohort was selected because Scots have a high risk of developing cancer, coronary heart diseases, stroke and dementia and the stability of the population is ideal for measuring how heritable and lifestyle factors could influence disease onset [52]. For the analyses, the authors relied on the Horvath and Hannum clocks, in which biological age is correlated with chronological age, together with Intrinsic Epigenetic Age Acceleration (IEAA) and Extrinsic Epigenetic Age Acceleration (EEAA). IEAA is not dependent on age-related changes in the cellular composition, while EEAA is based on the functional decline of the immune system. Risk factors were divided into four groups: (i) cognitive reserve (education and economic status); (ii) type 2 diabetes and high blood pressure; (iii) lifestyle factors (body mass index, smoking, HDL, cholesterol, total cholesterol; and (iv) genetics (AD polygenic risk score, AD family history and APOE). Although there was no link with IEAA, the results showed an existing correlation between cognitive reserve factors and IEAA. No correlation between genetic risk factors related to AD onset, such as family history or *APOE4* carrier status, has been detected (McCartney et al., 2018) (Table 1) [50].

Coninx et al. [37] developed cortical and hippocampal epigenetic clocks using murine models. In detail, epigenetic age investigations were carried out in two female mouse strains: C57BL/6J (B6 mice) and triple transgenic AD (AD mice) ranging from 3 months (young adults) to 15 months (aged adults). Genomic DNA, isolated from the cortex and hippocampus, was used to quantify DNAge^®^ a mouse epigenetic aging clock targeting 2031 age-associated CpG loci. Bisulfite-converted DNA libraries were sequenced on a HiSeq 1500 sequencer for >1000× coverage and aligned with bisulfite sequence data and methylation calling. The results demonstrated that there was an epigenetic age acceleration in AD mice compared to B6 mice, especially during early life, and this aspect was more marked in the cortex samples compared to hippocampus. These findings led the authors to hypothesize that early-life modifications in DNA methylation patterns could be responsible for the expression of genes related to aging and age-related diseases in adulthood. The authors also identified significant differentially methylated CpGs loci in genes critical for neurological functions or related to neurodegeneration and AD, highlighting the important role of epigenetic mechanisms underlying AD pathology or brain aging (Table 1) [37].

Grodstein et al. [53] applied four established epigenetic clocks (Hannum, Horvath, PhenoAge, GrimAge), together with a new Cortical clock, to assess DNA methylation age in DLPFCTX specimens of 721 participants with a mean age at death of 88 years. This aged cohort, without signs of dementia at the time of enrollment, was selected from the Religious Orders Study (ROS) and Rush Memory Aging Project (MAP). DNA methylation levels were assessed using the Illumina Infinium HumanMethylation450 (450 K) BeadChip and reported as a beta value (the ratio of the methylated probe intensity to the sum of methylated and unmethylated probe intensities). DNA methylation age from Hannum clock, Horvath clock, PhenoAge clock and GrimAge clock was calculated using an open-source software, while that from the Cortical clock was obtained using publiclyavailable code provided by the authors. The results showed that Hannum, Horvath, PhenoAge and especially Cortical clock ages were associated with a pathologic diagnosis of AD and Aβ load. Moreover, there was a notable association between Cortical age and global AD pathology. As regards the association between epigenetic clocks and clinical aging phenotypes, the authors found that epigenetic age was not related to clinical Alzheimer’s dementia. In contrast, there was an inverse correlation for the older GrimAge with lower odds of dementia and significantly better rates of cognitive decline over time for global cognition, semantic memory and working memory (Table 1) [53].

Milicic et al. [54] evaluated the relationship between five measures of age acceleration, established by DNA methylation patterns (DNAm age) and differences in AD-related neuroimaging phenotypes. For these investigations, two cohorts were selected: (i) Australian Imaging, Biomarkers and Lifestyle (AIBL, mean age 73.43 ± 6.99); (ii) Alzheimer’s Disease Neuroimaging Initiative (ADNI, mean age 73.9 ± 7.51). The AIBL cohort consisted of 373 subjects grouped as 240 cognitively unimpaired (CU), 69 mild cognitive impairment (MCI) and 64 Alzheimer’s (AD); the ADNI cohort consisted of 486 subjects grouped as 166 CU, 256 MCI and 64 AD. For both cohorts, data for PET imaging, MRI or cognition evaluation were collected. The Aβ PET status was considered Aβ-negative (Aβ-; <20 CL) or Aβ-positive (Aβ+; ≥20 CL). DNA methylation patterns were analyzed on blood samples using the Infinium HumanMethylation EPIC (850 K) BeadChip array. Horvath, Hannum, Phenoage, Zhang elastic net (EN) and Zhang Best Linear Unbiased Prediction (BLUP) were the epigenetic clocks employed to calculate DNAm age. The authors also utilized disproportionate biological age (DBAge, the residual from regressing biological age on chronological age) and difference in age (DiffAge, calculated by subtracting chronological age from biological age) as measures of age acceleration/deceleration. The results showed that in the AIBL cohort, there was a significant association between accelerating aging and hippocampal volume using the Hannum and Phenoage clocks. In the CU Aβ+ group, there was a significant association between hippocampal volume and accelerated aging using the Zhang EN, Hannum and Phenoage clocks. The same results were observed in the ADNI cohort, where the CU Aβ+ group showed a significant association between hippocampal volume and accelerated aging, using the Hannum clock. These results demonstrated that accelerated biological aging was associated with cross-sectional measures of brain volume (Table 1) [54].

Lynch et al. [55] evaluated Cortical clock age in association with cognitive status, Telomere Length (TL) and Mitochondrial DNA Copy Number (mtDNA-CN) in DLPFCTX tissues of 367 United States (US) non-Latino white participants of the Religious Orders Study (ROS) or the Rush Memory and Aging Project (MAP). In these cohorts, mean age at death was 88 years and 246 subjects were diagnosed with AD. TL and mtDNA-CN were bioinformatically estimated from Whole-Genome Sequencing (WGS) data. DNA methylation profiles from DLPFC tissues were generated using the Illumina Infinium HumanMethylation450 (450 K) BeadChip. The cortical clock was designed in postmortem cortical specimens to predict the chronological age from 347 CpG sites. The authors established a multimodal aging approach, creating a binary definition of accelerated biological aging based on Cortical clock age and mtDNA-CN. Accelerated Cortical clock age (CCage+) and non-accelerated Cortical clock age (CCage-) were combined with age-accelerated mtDNA-CN (mtDNACage+) and non-age accelerated mtDNA-CN (mtDNACage-). The combination of these binary biological age accelerations gave two predictors: (i) CCage+/mtDNACage+, which represented an individual who is age-accelerated on both clocks and (ii) CCage-/mtDNACage-, which represented an individual who is not age-accelerated on either the mtDNA-CN or the Cortical clock. The results showed that the Cortical clock was inversely associated with global cognition and that Cortical clock age was positively associated with four pathologic measures of AD (NIA-Reagan diagnosis, global AD pathology, Aβ aggregates and tau tangles). Additionally, mtDNA-CN was associated with global AD pathology and tau tangles. Furthermore, individuals with age acceleration on both measures CCage+/mtDNAC age+ had a stronger association with global cognition compared to either CCage+ or mtDNAC age+ alone, highlighting that age acceleration determined on multiple genomic measures might have a larger effect on AD-Related Disorders’ (ADRDs) pathogenesis (Table 1) [55].

Whitman et al. [56] examined the association between DunedinPACE and multiple measures of brain structure across three cohorts: (i) the Dunedin Study (770 subjects from New Zealand, mean age = 45 years), (ii) the Framingham Heart Study Offspring Cohort (FHS-OC; 903 subjects from Massachusetts, mean age = 63.76 years), and (iii) the Alzheimer’s Disease Neuroimaging Initiative (ADNI; 649 individuals; mean age = 75.41 years). For the Dunedin Study and ADNI cohorts, DNA methylation was measured from whole blood using Illumina Infinium MethylationEPIC BeadChip, while in the FHS-OC cohort, DNA methylation was measured from whole blood using Illumina Infinium HumanMethylation450 (450 K) BeadChip. The authors evaluated the associations between DunedinPACE with Total Brain Volume (TBV), Hippocampal Grey Matter Volume (HC), White Matter Hyperintensity (WMH), Mean Cortical Thickness (CT) and Total Cortical Surface Area (SA). In the ADNI cohort, the association between DunedinPACE and age-related changes in structural brain integrity was tested. The association between first- and second-generation epigenetic clocks (Horvath, Hannum, GrimAge, and PhenoAge) and structural brain integrity was assessed. The results showed that, in all three datasets, accelerated biological aging, reported as faster DunedinPACE, was related to lower TBV, lower HC and thinner cerebral cortex. Only in the Dunedin Study and in FHS-OC cohorts did people with faster DunedinPACE have a greater WMH volume. In the Dunedin Study, individuals with faster DunedinPACE had less total cortical surface area. In the ADNI cohort, faster DunedinPACE affected TBV, HC, WMH, CT and SA. When these results were compared with other epigenetic clocks, the only significant association was found between GrimAge and CT in the ADNI cohort, demonstrating that DunedinPACE and GrimAge had the strongest and most consistent associations with brain phenotypes (Table 1) [56].

Cruz-Gonzàlez et al. [57] tested the reliability of epigenetic clocks in different ethnic groups. Samples were obtained from a previous study called MAGENTA, consisting of 621 subjects (313 AD patients and 308 matched controls) representative of five cohorts from the US (African American, Cuban, white, Peruvian and Puerto Rican) with a mean age of 76 years old. This cohort was previously genotyped by Illumina Infinium Global Screening Array using standard-quality control filters. DNA methylation was assessed using the Illumina HumanMethylation EPICv2.0, while quality control and data normalization were performed using the openSeSAMe pipeline from the SeSAMe tools. Methylation clocks belonging to all generations were used (Horvath, Hannum, Zhang2019_EN, Zhang2019_BLUP and PhenoAge). In the white cohort, age predicted from DNAm age using the Horvath clock was strongly correlated with chronological age, while this correlation was lower in cohorts with African ancestry (Puerto Rican and African American). Compared to the Horvath clock, Hannum and Zhang2019_EN clocks showed a stronger correlation between methylation clocks and chronological age in all groups, while the PhenoAge clock showed a low correlation between DNAm age and chronological age. The authors also evaluated the ability of methylation clocks to identify accelerated aging and risk for AD. The results demonstrated that AD patients in the white cohort had greater age acceleration, as measured by the Horvath clock, than the controls, while the results were inconsistent when this comparison was applied in other ethnic groups. The authors hypothesized that several genetic factors could contribute to reducing the accuracy of the clocks; for example, the presence of variants that disrupt clock CpG sites prevents methylation, as does the presence of Methylation Quantitative Trait Loci (meQTLs) that influence clock CpG site methylation (Table 1) [57].

Savin et al. [58] measured the association between age acceleration and cognitive decline in 2296 US non-Hispanic white adults belonging to the Framingham Heart Study Offspring Cohort (FHS; mean age 25–101 years). In that cohort, 114 subjects were affected by AD. Cognitive decline in these individuals was evaluated across 23 years of neuropsychological-testing follow-up. DNA methylation was measured from whole-blood DNA samples and assays were performed with the Illumina Infinium HumanMethylation450 (450 K) BeadChip. Pace of aging in that cohort was measured using DunedinPACE, PhenoAge and GrimAge epigenetic clocks. The results showed that participants with a faster pace of aging had a worse average cognitive performance and more rapid cognitive decline during the follow-up. These data were confirmed when age acceleration was measured using PhenoAge and GrimAge epigenetic clocks. The authors also evaluated the sensitivity of associations between pace of aging and cognitive decline on the basis of ADRD risk factors: cognitive reserve, sex and *APOE4* carrier status. Individuals with better baseline cognitive functioning were more protected from risk associated with a faster pace of aging. Associations of pace of aging with the rate of cognitive decline were similar for men and women and for carriers and non-carriers of *APOE4* (Table 1) [58].

Bonham et al. [59] employed longitudinal modeling and structural neuroimaging analyses to test the hypothesis that accelerated epigenetic aging would predict AD progression. For this investigation, DNA methylation and structural neuroimaging data from 404 US participants of ADNI study were used. Among that cohort, 121 participants (mean age 75 years) were considered cognitively normal (CN), 236 (mean age 72 years) were diagnosed with MCI and 47 (mean age 74 years) were diagnosed with AD. DNA methylation data were obtained from blood samples using the Illumina InfiniumHuman MethylationEPIC V1 BeadChip Array, with a coverage of ~ 866,000 CpGs sites (illumina.com). Data were normalized using the wateRmelon R package and quality control procedure was performed. Epigenetic age was estimated using DNAmPhenoAge and DNAmGrimAge clocks. The results showed that in CN individuals, accelerated epigenetic age was related to progression to either MCI or AD, with cognitive decline independent of chronological age. Moreover, accelerated epigenetic age, measured with DNAmPhenoAge and DNAmGrimAge, was associated with cortical thinning in AD-relevant regions and WMH burden across the spectrum of normal aging to neurodegenerative disease. Interestingly, the relationship between epigenetic age and cortical thinning in AD-implicated regions was more relevant in CN participants, while the association between epigenetic age and WMH was greatest in AD patients. These findings demonstrated that advanced epigenetic age modulates the risk for AD and cognitive decline even in the early stages of disease (Table 1) [59].

Guo et al. [60] tried to identify a causal link that could elucidate the genetic etiology existing between AD and epigenetic clocks, along with AD in association with multivariate longevity-related phenotypes. The authors used a large-scale GWAS dataset consisting of publicly available GWAS summary statistics of neurodegenerative diseases (AD); four measures of epigenetic clock (GrimAge, PhenoAge, IEAA e HannumAge); and multivariate longevity. All participants had European ancestry and were grouped considering three main phenotypes: healthspan, parental healthspan and extreme longevity. The results showed an association between AD and GrimAge age acceleration, confirmed by the presence of a shared locus, rs78143120, novel for AD. The position of this locus has been established on chromosome 2 and was also proposed as a novel risk locus for the onset of AD. Colocalization analysis, performed to detect distinct AD genomic loci associated with longevity, displayed a correlation between rs12691088 and exceptional longevity (Table 1) [60].

**Table 1:** Main findings achieved through the evaluation of epigenetic clocks’ efficacy in studies involving AD subjects. AD: Alzheimer’s disease; DLPFCTX: dorsolateral prefrontal cortex; BMI: body mass index; HDL: High-density lipoprotein; TL: telomere length; mtDNA-CN: mitochondrial DNA copy number; Aβ: amyloid beta peptide; DunedinPACE: Dunedin Pace of Aging calculated from the epigenome; HC: hippocampal grey matter volume; CT: cortical thickness; MCI: mild cognitive impairment; GWAS: Genome-Wide Association Study; IEAA: intrinsic epigenetic age acceleration.

References	Samples	Epigenetic Clocks	Tissues	Aim	Main Findings
Levine et al. [48]	700 subjects	Horvath	DLPFCTX	Association between epigenetic age, AD and neuropathological markers	Epigenetic age acceleration was linked to neuritic plaques, β-amyloid load and neurofibrillary status
Lu et al. [49]	1163 subjects	Horvath	Brain biopsies	Evaluation of aging rate and investigation of genetic loci associated with accelerated aging in several brain regions	Epigenetic age correlated with chronological age. Genes associated with cognitive decline, dementia and AD have been detected
McCartney et al. [50]	5100 subjects	Horvath and Hannum	Blood	Detection of a link between AD risk factors and age acceleration	Age acceleration was linked with BMI, total cholesterol to HDL ratios, socioeconomic status, high blood pressure and smoking levels
Coninx et al. [37]	Mice	Cortical and Hippocampal	Cortex and hippocampus	Validation of two tissue-specific epigenetic clocks to estimate epigenetic age acceleration	Epigenetic age acceleration was more noticeable in brain cortex in comparison with hippocampus
Grodstein et al. [53]	721 subjects	Hannum, Horvath, PhenoAge, GrimAge, Cortical Age	DLPFCTX	Association between epigenetic clocks, brain disease and clinical aging phenotypes	Hannum, Horvath, PhenoAge and Cortical clock were associated with diagnosis of AD. Otherwise, there was no association between epigenetic clocks and clinical aging phenotypes
Milicic et al. [54]	859 subjects	Horvath, Hannum, PhenoAge, Zhang EN, Zhang BLUP	Blood	Establishing a relationship between five measure of age acceleration and AD-related neuroimaging phenotypes	The use of Hannum, PhenoAge and Zhang EN demonstrated an important association between accelerated aging and hippocampal volume. Accelerated aging was also associated with cross-sectional measure of brain volume
Lynch et al. [55]	367 subjects	Cortical	DLPFCTX	Association between cortical clock age and cognitive status, TL and mtDNA- CN	Cortical clock was inversely related to global cognition and positively associated with Aβ aggregates and tau tangles’ deposition. mtDNA-CN levels reflected global AD pathology and tau tangles
Whitman et al. [56]	2322 subjects	DunedinPACE, GrimAge	Blood	Association between DunedinPACE and different measures of brain structure	Accelerated aging, reported as faster DunedinPACE, was related to total brain volume, lower HC and thinner cerebral cortex
Cruz- Gonzàlez et al. [57]	621 subjects	Horvath, Hannum, Zhang EN, Zhang BLUP, PhenoAge	Blood	Reliability of epigenetic clocks to detect AD risk in different ethnic groups	A strong relationship between epigenetic age and chronological age, with all generations of epigenetic clocks, was detected in the AD white cohort, while in the African ancestry cohort, this correlation was weaker
Savin et al. [58]	2296 subjects	PhenoAge, GrimAge and DunedinPACE	Blood	Association between age acceleration and cognitive decline	Participants with a faster pace of aging showed a rapid cognitive decline
Bonham et al. [59]	404 subjects	PhenoAge, GrimAge	Blood	Recognition of a link between accelerated epigenetic aging and AD prediction and progression	Accelerated epigenetic aging was associated with progression of MCI or AD
Guo et al. [60]	63926 subjects, data obtained from GWAS Dataset	GrimAge, PhenoAge, IEAA, Hannum	Blood	Estimation of a causal relationship between epigenetic clocks and AD	There is an association between AD and GrimAge age acceleration

## 4. Discussion

### 4.1. Epigenetic Clocks: Advantages and Challenges in Measuring Biological Aging in Neurodegenerative Disorders and Alzheimer’s Disease

Aging represents a progressive and natural biological process in which the decline of multiple organs over time predisposes individuals to environmental stressors, increasing their susceptibility to diseases [61]. Aging is the main contributor to the onset of cognitive disturbances and neurodegenerative diseases, including AD, the most common form of dementia, with a constant increasing prevalence worldwide [62]. For that reason, the scientific community is making numerous efforts to discover the mechanisms that orchestrate the adverse phenotypic alterations linked to the aging process [4].

Aging could be directly responsible for the alterations in the expression of several genes, caused by prolonged exposure to environmental stressors, together with epigenetic modifications. Among the epigenetic changes, DNA methylation plays a crucial role due to its ability to predict the aging state and measure biological age, thus helping to detect all the aspects linked to healthy aging [63].

This feature is particularly important in the landscape of neurodegenerative diseases and, in particular, for AD, for which a prompt diagnosis and effective therapeutic treatments are still lacking. Although several biomarkers for dementia are currently available, the high costs, the invasiveness of the methods and the different interpretations of the results by specialists make these methods unsuitable for clinical practice [64,65].

Nowadays, the idea that fluctuations in DNA methylation levels could serve as an “epigenetic clock” has gained traction, revealing that this parameter might work as an accurate biomarker to estimate “biological age” and to predict adverse age-related changes [29]. However, more studies are needed to understand the causal connection between DNA methylation and aging, which is a complex process involving multiple factors such as loss of proteostasis, mitochondrial dysfunction, stem cell exhaustion and immunosenescence [66].

Until now, studies on epigenetic clocks involved heterogeneous subgroups and different tissue samples, thus limiting the representativeness and the statistical power of the findings. These limitations might hamper the ability to establish a causal relationship between epigenetic clocks and disease progression, weakening the efficacy of this parameter for the application in clinical practice. Another concern derives from the difficulty to quantify biological aging in diverse populations due to the lack of sociodemographic and socioeconomic characteristics, which play a crucial role in both DNA methylation variations and healthy aging. The acquisition of predictive models involving numerous social groups in the training data could improve the sensitivity and reliability of epigenetic clocks [67].

Significant aberrant DNA methylation patterns have been found in neurodegenerative diseases, where disrupted CpG methylation sites have been reported in a set of genes involved in many cellular pathways. DNA methylation plays a remarkable role in the onset of AD, and methylation in *APP*, Apolipoprotein E (*APOE*), *PSEN1*, *PSEN2* and other aging-related genes seems to actively contribute to the manifestation of this disorder [68]. For example, hypermethylation of the *APOE* promoter region has been detected in the prefrontal cortex of AD patients, causing decreasing levels of circulating ApoE [69]. Epigenetic modifications in the promoter region of *PSEN1* are responsible for an increased expression of this gene, a condition considered a high-risk factor for AD. Indeed, it has been demonstrated that the PSEN1 gene remains almost methylated and its activity repressed after development, while hypomethylation events have been frequently associated with its elevated expression in AD patients [70].

On the contrary, genes whose activity is considered “protective” for the risk to develop AD, such as nuclear export protein (NEP) and ankyrin 1 (ANK1), have been found to be highly methylated. These events could be responsible for a reduced gene expression, resulting in an increase in β-amyloid deposition [71]. Moreover, remarkably higher levels of 5-Hydroxymethylcytosine (5 hmC) in the middle frontal gyrus and in the middle temporal gyrus of AD brains have been detected, thus corroborating the existence of a positive correlation between high 5 hmC levels and NFTs’ aggregation [72].

### 4.2. Epigenetic Clocks as Biomarkers to Monitor Healthy Aging and Cognitive Decline

In this review, we performed an extensive search of the literature in order to explore the relationship between DNA methylation and epigenetic clocks and how epigenetic alterations could predict the risk of developing cognitive decline and AD, revealing the main mechanisms through which DNA methylation regulates the aging process. Epigenetic clocks might be a useful tool to monitor aging in a tissue-specific manner and be used in a wide variety of samples, including cerebral cortex, brain tissues and blood [32]. Although some epigenetic clocks, such as Horvath, have been defined as “pan-tissue clock”, other epigenetic clocks have been designed for peripheral blood samples, and further calibrations have been performed to gather a multi-tissue predictor for age [30]. Indeed, methylation levels diverge among different tissues and this inconsistency may be reflected in methylation profiles. Furthermore, comparing results obtained from post-mortem brain tissue analysis and blood samples may be difficult because other environmental factors may influence methylation patterns [73]. However, even if blood does not provide a complete estimate of the changes found in the brain, it could give information on the methylation status of the target tissue, thus representing the peripheral response to such fluctuations. For this reason, further investigations on epigenetic changes in different samples are needed to glean the onset and progression of AD at different stages with the aim of identifying prognostic/diagnostic biomarkers. Levine et al. demonstrated the efficacy of measuring epigenetic aging in DLPFC samples to detect the risk of developing cognitive decline. Because the analysis was conducted at a single time point in post-mortem samples, it was impossible to observe whether changes in DNAm age were linked to cognitive decline or AD. However, the use of neuropathological tools, together with the measurement of multiple cognitive functioning domains, revealed a strong association between DNAm Age and five neuropathological hallmarks of AD (amyloid load, neuritic plaques, diffuse plaques, NFT, and overall tangle score), along with the detection of pleiotropic genetic loci that could influence epigenetic age acceleration and cognitive traits. The early phase of dementia is usually characterized by memory impairment and cognitive decline; for that, reason the ability of epigenetic clocks in monitoring these episodes could provide an opportunity for a prompt intervention in the transition from healthy aging to MCI and to dementia [48]. The mechanism through which genetic loci with pleiotropic effects could influence epigenetic age acceleration, neuropathological variables, and cognitive traits has also been highlighted in the work of Lu et al. Gene sets identified by GWAS data of PFCTX samples, examined for epigenetic aging and an estimated proportion of neurons, were significantly enriched with genes associated with cognitive decline, dementia and AD. In particular, the rs2054847 variant located in 17q11.2 locus was significantly associated with the expression levels of the *EFCAB5* gene, involved in processes such as Ca2+ signaling, synaptogenesis, dendritic arborization and cell survival. Furthermore, the rs11296960 variant located near the *ECE1* gene, encoding an Aβ-degrading enzyme in the brain, was related to epigenetic age acceleration, and decreased levels of *ECE1* were associated with reduced Aβ clearance and increased plaque deposition. The discovery that both biomarkers of brain aging were correlated with age-relating phenotypes provided new biological insights into the mechanisms through which the genetic architecture of epigenetic and neuronal aging could influence human brain regions [49]. The advent of analysis building on the employment of epigenetic clocks gave the opportunity to investigate the associations and shared genetic etiology between neurodegenerative diseases and epigenetic age acceleration, as well as neurodegenerative diseases and multivariate longevity. Guo et al. performed large-scale GWAS summary statistics taking into account four measures of epigenetic age (GrimAge, PhenoAge, IEAA, and HannumAge), multivariate longevity (healthspan, lifespan, and exceptional longevity) and multiple neurodegenerative diseases. This approach led to the identification of a novel rs78143120 variant (located on the *AC093375.1* gene) associated with AD and GrimAge age acceleration, together with the rs12691088 variant (located on the *APOC1* gene) associated with AD and exceptional longevity. This study demonstrated the importance of investigating relevant phenotypes of aging and their genetic associations with neurodegenerative disease phenotypes. Indeed, some genes in AD-associated loci and GrimAge age acceleration might be involved in crucial mechanisms (Aβ plaque deposition, microglial accumulation around Aβ plaques and cognitive decline) that determine the onset of AD [60]. It has been proposed that lifestyle risk factors for AD, such as body mass index (BMI), smoking and cholesterol could play a crucial role in the acceleration of aging process. In line with this premise, McCartney et al. identified significant associations between IEEA and EEAA and BMI, cholesterol and smoking levels. These results demonstrated that epigenetic clocks could be a robust predictor of chronological age, as well as the greatest risk factor for AD in advanced age. Furthermore, since epigenetic alterations are chemically reversible, therapeutical measures aiming to modify such risk factors might reverse these modifications, reducing the risk profile for AD and the rate of biological aging [50]. The association between epigenetic aging and modifications in some brain regions has also been confirmed in animal models due to limited access to human brain tissues. Coninx et al. found accelerated epigenetic age in brain regions of the AD mouse model compared to non-pathological mice. These modifications were marked in AD mice even in early life. Moreover, AD mice prematurely started to show accumulation of Aβ oligomers in the cortex, along with DNA methylation changes at some CpG-sites (*Hdgfl2*, *Calb2* and *Srcin1*) involved in neurological functions and synaptic plasticity. Although designed for animals, epigenetic clocks were able to examine the timing of epigenetic age modification during the disease process, identifying accelerated aging factors associated with AD [37]. DNA methylation status in DLPFCTX specimens has also been investigated by Grodstein et al., who exploited the simultaneous use of traditional epigenetic clocks (Hannum, Horvath, PhenoAge, GrimAge) and a novel Cortical clock. The results showed that epigenetic clocks trained in blood samples did not predict most neurologic phenotypes, while Cortical clock age was strongly correlated with AD pathologic diagnosis and Aβ load [53]. The same results were obtained by Milicic et al., who used multiple methodologies for calculating age acceleration to assess the relationship between DNAm age and AD-related neuroimaging phenotypes. Although there was no association between accelerated aging and brain Aβ load or cognitive decline, a significant association was observed between age acceleration using the Hannum clock and cross-sectional hippocampal volume in the selected cohorts [54]. These findings emphasized that the limited results of the studies might reflect the limitations of existing clocks themselves, highlighting the critical need for bespoke tailoring clocks of brain-aging-targeting CpG sites and those conserved across brain and other tissues, which could better represent aging processes [53,54]. To overcome these limitations, Lynch et al. pooled multiple genomic measures of aging (Cortical clock, TL and mtDNA-CN) to create a binary variable predicting accelerated aging and its association with clinical dementia and neuropathological traits. In this case, not only was the Cortical clock inversely related to global cognition and positively related to pathologic measures of AD, but the strength of the association was more pronounced when the Cortical clock and mtDNA-CN acceleration (expressed as CCage+/mtDNAC age+) were used in combination [55]. As previously stated in the Introduction, mitochondria regulate both cellular metabolism and apoptosis; therefore, mitochondrial dysfunction plays a crucial role in AD pathogenesis. Indeed, a DNAm increase in the promoter of the *ELOVL2* gene has been reported in the early stage of AD compared to controls, resulting in increased endoplasmic reticulum stress and mitochondrial dysfunction. Interestingly, the methylation levels of the *ELOVL2* gene have also been associated with p-tau protein deposits in human hippocampal regions, suggesting that this gene may be involved in the development and progression of AD [74]. Moreover, demethylation of the Displacement Loop (D-loop) region, which regulates mtDNA transcription, has been found in AD animal models and human samples. On the other hand, genes encoding 12S rRNA, Cytochrome B (CYTB) and Cytochrome C Oxidase Subunit II (COX II) were hypermethylated with decreased mtDNA-CN [75]. These findings demonstrated that there is an urgent need to use multiple parameters for a clear measurement of the aging process. Implementation of the analyses using a combination of mtDNA-CN and Cortical clock age could help to identify a stronger correlation between age acceleration and cognitive and neuropathological outcomes.

Some studies relying on multiple geroscience biomarkers (telomere length and erosion, epigenetic clocks and biomarker composites) found low agreement between different measures of biological aging [76]. These results suggested that sometimes the combination of several approaches might not be ideal because each application might not evaluate the same aspects of the aging process. For that reason, there is a need for further systematic evaluation of a way to integrate multiple biomarkers in order to develop reliable measures of biological aging [76].

### 4.3. Epigenetic Clocks: Limitations and Future Perspective

Aging is indeed a whole-body process; therefore, the use of an epigenetic index of an individual’s pace of biological aging, obtained by monitoring multi-organ decline, could provide more information on age acceleration associated with dementia and measures of structural brain integrity. This hypothesis has been confirmed by Whitman et al., who examined the association between DunedinPACE and multiple measures of brain structure across three cohorts ranging from middle-aged to elderly individuals. In all three datasets, a faster DunedinPACE was related to lower TBV, lower HC and a thinner cerebral cortex. Moreover, in the ADNI cohort, faster DunedinPACE affected TBV, HC, WMH, CT and SA. When the authors used other epigenetic clocks to compare their results, the only significant association was found between GrimAge and CT in the ADNI cohort [56]. The discrepancy might be caused by the limited ability of aging biomarkers, trained only on chronological age, to detect age-related health outcomes. This aspect could be improved by using biomarkers representative of biological aging, such as DunedinPACE [77,78]. These data support the geroscience hypothesis, emphasizing that progressive body decline reflects the decline in structural brain integrity during middle and old age [79]. The main limitation in the use of methylation clocks is the difficulty in corroborating data with the same accuracy across different ethnic groups that are often underrepresented in genetic and genomic databases. In the work of Gonzalez et al. [45] epigenetic clocks failed to quantify the rate of aging from DNA methylation patterns, as well as the risk of AD, in genetically admixed individuals. The authors hypothesized that this discrepancy could be explained by the fact that meQTLs, genetic variants that associate with the methylation level of a CpG site across individuals, could influence the outcomes of epigenetic clocks. MeQTL could influence methylation levels via many regulatory mechanisms, such as disruption of CpGs sites and effects on transcription factor binding. These variants have been frequently detected in African ancestry individuals, thus contributing to the reduction in the clocks’ accuracy. For these reasons, epigenetic clocks lacking CpGs sites with meQTLs, such as DuedinPACE clock, might ensure a more reliable performance in an admixed cohort.

It has been proposed that a low diversity of samples used to investigate epigenetic predictors might provide poor outcomes as a consequence of demographic variables including race, ethnicity and country of origin. One way to overcome this gap is to transfer the learning framework in order to better adapt existing epigenetic clocks to underrepresented populations using shared knowledge from diverse datasets and improving the strength of performances [80].

Furthermore, including individuals of multiple genetic ancestries (such as African American individuals) in the training cohort could improve the detection of age acceleration, increasing methylation clocks’ performance [57]. Although previous studies have shown that older adults with a faster pace of aging exhibit brain features typical of neurological disorders and AD, it is not yet fully understood how cognitive decline reflects specific brain processes in older adults. To this end, Savin et al. integrated data from previous studies using epigenetic clocks to measure cognitive decline in a cohort across two decades of neuropsychological testing follow-up. Individuals with a faster pace of biological aging at baseline, measured with the DunedinPACE, PhenoAge and GrimAge epigenetic clocks, showed poorer cognitive performances and a quicker cognitive decline at follow-up based on the ADRD risk factors. In contrast, a faster pace of aging was less harmful among older adults with higher cognitive tasks at baseline, suggesting not only that the pace of aging might be related to features of the brain promoting cognitive resilience to neuropathology, but also that systemic biological aging could play a key role in the aging of the brain [58]. These findings highlight the potential of epigenetic clocks to serve as tools to prevent the adverse events of unhealthy aging while also serving as a biomarker to detect cognitive decline early and ensure timely therapeutic interventions.

The study also emphasized the importance to frequently monitor cognitive decline among the investigated cohorts to obtain reliable data. In this case, all participants underwent annual standardized neuropsychological examinations, beginning from 19 to 47 years from the study’s baseline and extending over 24 years of follow-up. Evidence that an accelerated pace of aging is indicative of future dementia risk might be the result of the repeated examinations performed by the authors during the study, thus supporting the need for a recurrent application of the epigenetic clocks to promptly catch all the hallmarks related to cognitive decline [58].

The advent of longitudinal modeling combined with structural neuroimaging analysis has enabled a better use of blood-based epigenetic clocks to detect the relationship between accelerated epigenetic aging and AD pathophysiology. Using survival analyses, Bonham et al. found that accelerated epigenetic age was associated with progression to MCI or AD, independent of chronological age, among CN individuals. This evidence highlighted that modifications promoted by biological aging contribute significantly to the development and progression of AD. Accelerated epigenetic age based on DNAmPhenoAge and DNAmGrimAge was associated with cortical thinning in AD-relevant regions and WMH burden across the spectrum of normal aging to neurodegenerative disease. These outcomes suggested that epigenetic age could contribute to increase the AD risk and that epigenetic clocks might offer a precious contribution to detect pathological changes among CN individuals before the onset of symptoms. This perspective appears very promising because current measures able to identify common markers of AD, such as Aβ plaques, rely on expensive and invasive technologies (PET scan or lumbar puncture). In contrast, the use of less invasive and cost-effective procedures, such as epigenetic clocks, in readily available samples such as blood could have a high social and economic impact for the early detection of age-related neurodegeneration and AD. This study also highlights the need to employ more epigenetic clocks capable of capturing more AD-related risk factors. Indeed, DNAmPhenoAge was found to be strongly associated with disease progression, while DNAmGrimAge was found to be more sensitive for the identification of neuroimaging biomarkers [59].

## 5. Conclusions

This review summarized the implications and the utility of using epigenetic clocks, applied to blood or different tissues, as a biomarker to assess the association between age acceleration and disease risk.

Studies on global DNA methylation changes have provided insights into the aging process, emphasizing how DNA methylation could serve as a biomarker to evaluate the driving force of this process. Moreover, the detection of target genes modified by DNA methylation-related regulatory elements in aging individuals could be highly informative for understanding the signaling pathways mediating disease-related alterations.

It is important to better investigate the aging process and its associated molecular mechanisms in order to select suitable epigenetic clocks in the context of a specific disorder. Epigenetic clocks able to simultaneously estimate brain and biological ages could provide valuable data for interventions aimed at preventing or slowing cognitive decline and the onset of AD. The complexity and heterogeneity of these measurements require the use of multiple parameters in order to detect key mechanisms of DNA methylation levels indicative of age-related decline. Data obtained by this approach could lead to the development of promising clinical trials that could evaluate new epigenetic therapies directed against neurodegenerative diseases, such as AD.

## Data Availability

No new data were created or analyzed in this study.

## Abbreviations

The following abbreviations are used in this manuscript:

CpG	Cytosine-phosphate-guanine
AD	Alzheimer’s Disease
DNA	Deoxyribonucleic Acid
ROS	Reactive Oxygen Species
MMR	Mismatch Repair
BER	Base Excision Repair
MAPK	Mitogen-Activated Protein Kinase
METTL3	Methyl Transferase-like 3
METTL14	Methyl Transferase-like 14
WTAP	Wilm’s Tumor-1-Associated Protein
m6A	N6-methyladenosine
tRNA	RNA transfer
rRNA	ribosomal RNA
ATP	Adenosine Triphosphate
OXPHOS	Oxidative Phosphorylation
IgG	Immunoglobulin G
IgA	Immunoglobulin A
DNMTs	DNA Methyltransferases
SAM	S-Adenosyl-Methionine
DunedinPoAm38	Dunedin Pace of Aging Methylation
DunedinPACE	Dunedin Pace of Aging calculated from the Epigenome
CRP	C-Reactive Protein
NFTs	Neurofibrillary Tangles
APP	Amyloid Precursor Protein
MAPT	Microtubule Associated Protein Tau
Ach	Acetylcholine
5hmC	5-hydroxymethylcytosine
Aβ	Amyloid-β
NLRP3	NOD-like Receptor Protein 3
NF-kB	Nuclear Factor Kappa B
SAD	Sporadic Alzheimer’s Disease
FAD	Familial Alzheimer’s Disease
EOAD	Early-Onset Alzheimer’s Disease
LOAD	Late-Onset Alzheimer’s Disease
PSEN1	Presenilin 1
PSEN2	Presenilin 2
EM	Episodic Memory
WM	Working Memory
SM	Semantic Memory
PO	Perceptual Orientation
PS	Perceptual Speed
GCF	Global Cognitive Functioning
GCTA	Genome-Wide Complex Trait Analyses
PFCTX	Prefrontal Cortex
DLPFCTX	Dorsolateral Prefrontal Cortex
CRBLM	Cerebellum
FCTX	Frontal Cortex
PONS	Pons
TCTX	Temporal Cortex
GWAS	Genome-Wide Association Study
CETS	Cell Epigenotype Specific
DNAm	DNA Methylation
IEAA	Intrinsic Epigenetic Age Acceleration
EEAA	Extrinsic Epigenetic Age Acceleration
HDL	High-Density Lipoprotein
APOE	Apolipoprotein E
ROS	Religious Orders Study
MAP	Rush Memory Aging Project
AIBL	Australians Imaging Biomarkers and Lifestyle
ADNI	Alzheimer’s Disease Neuroimaging initiative
CU	Cognitively Unimpaired
MCI	Mild Cognitive Impairment
PET	Positron Emission Tomography
MRI	Magnetic Resonance Imaging
EN	Zhang Elastic Net
BLUP	Zhang Best Linear Unbiased Prediction
DBAge	Disproportionate Biological Age
DiffAge	Difference in Age
TL	Telomere length
mtDNACN	Mitochondrial DNA Copy Number
US	United States
WGS	Whole-Genome Sequencing
NIA	National Institute on Aging
(CCage+)	Accelerated Cortical Clock Age
(CCage-)	Non-Accelerated Cortical Clock Age
(mtDNACage+)	Age-Accelerated mtDNA-CN
(mtDNACage-)	Non-Age-Accelerated mtDNA-CN
ADRDs	AD-Related Disorders
FSH-OC	Framingham Heart Study Offspring Cohort
TBV	Total Brain Volume
HC	Hippocampal Grey Matter Hyperintensity
SA	Total Cortical Surface Area
meQTLs	Methylation Quantitative Trait Loci
CN	Cognitively Normal
BMI	Body Mass Index
D-loop	Displacement loop
CYTB	Cytochrome b
COX II	Cytochrome C Oxidase Subunit II
